# Supplementary material for: Are carbon emissions trading and green financial instruments synergistic? -Comprehensive quantitative research based on content analysis
Source: PLoS One. 2024 Mar 7;19(3):e0298601. doi: 10.1371/journal.pone.0298601 (PMC10919601; doi:10.1371/journal.pone.0298601)
Supplement: S1 Data — (ZIP) [file pone.0298601.s001.zip › Data/地级市Z_F工作报告.zip!/╔╜╢1⁄2/╚╒╒╒╩╨/└┤╫╘╣1⁄2╓┌║┼í╛┬φ┐╦╩2╛▌═°í┐_2005.docx]

各位代表：

现在，我代表市人民政府，向大会作政府工作报告，请予审议，并请市政协各位委员和其他列席会议的同志提出意见。

一、2004年政府工作回顾

2004年是我市经济社会快速发展、城市面貌深刻变化、各方面工作亮点凸显的一年。面对国家加强宏观调控的新形势，各级政府在市委的正确领导下，团结带领全市人民，坚持以邓小平理论和"三个代表"重要思想为指导，牢固树立和认真落实科学发展观，坚定不移地推进跨越发展，圆满完成了市十五届人大二次会议确定的各项任务。全年生产总值初步核算实现387.78亿元，比上年增长17.2%；完成地方财政收入11.23亿元，按可比口径增长34.1%，两项指标增幅均创1996年以来最高水平。社会消费品零售总额增长16.6%，增幅创1997年以来新高。一、二、三产业增加值分别增长7.2%、24%和13.6%，三次产业比例由上年的18.5:44.6:36.9调整为16.1:49.3:34.6。特别令人鼓舞的是，经过全市人民的不懈努力，我市"十五"计划的主要指标提前一年基本完成，这是我市跨越发展进程中取得的又一重大成就！

（一）工业发展取得历史性突破。增长速度明显加快。规模以上工业增加值首次突破100亿元，实现销售收入333.3亿元，分别增长40%和63.5%，工业用电量增长37.9%，这三项指标增幅均居全省前两位。骨干企业快速膨胀。全市销售收入过亿元的企业达到63家、新增27家，过10亿元的达到8家、新增4家。特别令人欣喜的是，2004年五征集团销售收入达到40.9亿元，增长78%，实现了连续六年高速增长；2003年当年开工、当年投产的日照钢铁厂，2004年销售收入就突破40亿元，再创了辉煌。这两家企业奇迹般的发展，结束了我市没有销售收入过40亿元企业的历史。发展后劲不断增强。全社会固定资产投资完成183.68亿元，增长40%，在建投资过亿元的项目14个、过千万元的169个。

（二）农村经济跃上新台阶。农业生产全面丰收。主要农作物产量均实现了增长，粮食单产、总产分别增长7.8%和7.6%，水产品产量增长14.3%。农村经济结构进一步优化。各类经济作物面积发展到160万亩，粮经比例达到49:51；畜牧业规模化养殖比重提高到45%；水产品工厂化养殖面积达到35万平方米，比上年增加10万平方米。产业化水平不断提高。各类产业化经营组织发展到1341个，龙头企业达到928家，其中销售收入过亿元的11家。农民收入大幅增长。人均纯收入达到3695元，增长11.8%，创近年来的新高。党在农村的各项政策全面落实。农民负担明显减轻，农业生产条件不断改善，农民生产积极性进一步提高。

（三）港口经济迅猛崛起。港口建设实现新突破。修订完善了港口总体规划，确立了建设日照、岚山两个亿吨大港的目标，先后开工建设投资过亿元的重点工程12项，总投资超过60亿元。日照港区25万吨矿石码头、集装箱码头1号泊位等5项工程已建成投产。全年港口货物吞吐量突破5000万吨大关，今年有望超过7000万吨。临港工业发展迅速。实现销售收入130亿元，占规模以上工业销售收入的39%，比上年翻了一番多，今年有望再翻一番。全市8个销售收入过10亿元的企业中，有7个是临港企业。港口物流快速增长。日照港与中石化合资12.6亿元建设30万吨级原油码头及中石化投资20亿元建设输油管线项目已签订合作协议，投产后年吞吐量可达2000万吨。海上客运增势强劲，日照至平泽航线出入境旅客超过7万人次，比上年增加5.4万人。我市发展港口经济的做法得到省政府领导的高度评价。

（四）特色经济成为新亮点。一是旅游经济快速发展。全年旅游人数和旅游收入分别增长48.4%和56%，增幅居全省第一位和第二位。特别是"渔家乐"民俗游成为日照独具特色的旅游品牌，目前已发展到12个村、3.6万张床位，王家皂、桃花岛、任家台3个村被国家旅游总局命名为"全国农业旅游示范点"。二是大学经济持续发展。大学科技园累计完成投资19亿元、建筑面积140万平方米，已有5所高校开学招生，在校大学生达到3.5万人。三是体育经济方兴未艾。去年，我们成功地承办了全国沙滩排球赛、帆船锦标赛等12项省级以上体育赛事，世帆赛基地被确定为"国家水上运动训练基地"和29届奥运会帆船帆板赛前集训场地。经过积极争取，我市在获得世界帆船锦标赛承办权的基础上，又获得了世界帆板锦标赛承办权，并将第十一届全运会的6个比赛项目争取到日照举办，使我市成为继济南、青岛之后第三个承办比赛项目最多的城市。今年将有世界帆船、帆板两项国际赛事和11项省级以上体育赛事在日照举办。四是房地产业保持了强劲的发展势头。开发投资和商品房销售额分别增长50%和60%，有60%以上的房产被外地居民购买。与此同时，商贸流通等服务业繁荣活跃，利群商厦、银座购物广场等一批大型商场和超市相继建成营业，城市服务业水平和整体功能得到有力提升。

（五）对外开放迈出新步伐。招商引资成效明显。全年合同外商投资2.31亿美元，实际外商投资1.07亿美元，均比上年翻一番。招引市外投资项目2146个，到位资金110.9亿元。投资115亿元的超超临界电厂项目已签订建设协议书，厂址初步敲定；投资48亿元的日照电厂二期工程项目建议书已上报国家发改委；投资4.8亿元的60万吨焦化项目一期工程建成投产；比利时百瑞集团投资5000万欧元的东升地毯一期工程开工建设，法国液化空气集团投资2990万美元的液化空气制造项目批准设立，实现了对欧招商的重大突破。对外贸易快速增长。全年完成进出口总值18.43亿美元，其中出口10.82亿美元，分别增长42.8%和38.6%；完成加工贸易出口额1.92亿美元，增长59%，高于一般贸易增幅24个百分点；出口创汇过千万美元的企业达到14家，比上年增加9家，市水产集团出口创汇超过1亿美元；实现海关税收30.6亿元，增长87.5%，其中海关代征税增长99.4%。开放环境得到优化。市行政服务大厅正式启用，有70%的行政审批项目纳入大厅集中办理，受到外商和企业的普遍好评。

（六）接轨青岛、融入半岛工作取得实质性进展。一是我们与青岛市政府签订了加强交流合作的框架协议，初步形成了两市领导、企业和各界人士互动的局面。我市有100个部门和单位与青岛对口单位建立了联系，各区县分别与青岛市相关区市结成友好对子，青岛来我市的回访交流活动达到90批1329人次。二是产业接轨成效明显。全年共从青岛引进项目97个，总投资28.37亿元，已建成投产37个，其中利群集团投资5000万元在我市建设的利群商厦已建成营业，青啤集团投资1.8亿元对日照公司搬迁扩建正在实施。我市农产品在青岛的市场份额进一步扩大，成为青岛市重要的"菜园子"之一。三是我市许多经济增长指标在半岛8城市中的位次明显前移，其中工业增加值、销售收入增幅居第1位，社会消费品零售总额、进出口总值增幅居第2位，地方财政收入增幅居第3位。我市接轨青岛、融入半岛的做法被省领导批转有关市参阅。

（七）城市面貌发生崭新变化。城市规划工作得到加强。委托中国城市规划院进行了城市发展战略研究，开展了新一轮城市总体规划修编，详规覆盖率由上年的65%提高到72%。建设和管理水平明显提高。开工建设了16项城建重点工程，城建总投资18亿元，是上年的3.3倍，建成了世帆赛基地、山海路、迎宾路、日照海洋馆等一批精品工程，其中世帆赛基地被国家体育总局领导誉为"亚洲第一、世界领先"，成为展示日照对外形象的重要窗口。城市环境更加优美。以创建国家卫生城市和国家环保模范城市为载体，深入开展了以"绿化、美化、净化"为重点的城市环境综合整治活动，对市区8条城市道路进行了维护罩面，对26条道路进行了综合整治，对51条道路的人行道进行了高标准改造铺装。新增绿化面积270万平方米，是上年的3.4倍，大绿量、花园式、生态化的海滨城市特色进一步显现。2004年我市获得了山东省城市环境综合整治第二名，被授予山东省适宜人居环境奖，省政府在我市召开现场会推广了我们的做法。

（八）社会事业全面发展。一是"科教兴市"战略深入实施。新增省级高新技术企业13家，高新技术产业产值增长40.5%，专利申请量增长97.3%，新创山东名牌产品3个。"普九"水平不断巩固提高，高中段教育普及率比上年提高15个百分点，职业教育、成人教育年培训65万人次。二是可持续发展成效明显。全面实施生态市建设规划，大力发展循环经济，城市环境质量居全省第一，2004年我市被确定为创建国家循环经济示范市。人口和计划生育目标管理被省委、省政府授予一等奖。土地、海洋、森林资源保护工作进一步加强，耕地总量保持动态平衡。三是各项事业取得新进步。就业和再就业工作得到加强，社会保障体系进一步完善。文化事业繁荣活跃，成功地举办了"海曲汉韵"文物陈列展、赴韩国平泽文化艺术交流和日照农民画赴法国、马来西亚展出等一系列活动，广电中心建成启用。民兵预备役训练基地基本完工。慈善总会挂牌成立。疾病预防控制和医疗救治"两个体系"建设走在全省前列，中医院门诊大楼投入使用。四是精神文明和民主法制建设得到加强。开展了文明城市创建活动，重视加强未成年人思想道德建设。农村民主政治建设、村务公开的经验在全省推广。新闻出版、民族宗教、外事侨务、对台事务、统计、物价、人防、老龄、史志、档案、气象、残疾人事业等也都取得了新的成绩。

（九）日照的对外形象明显提升。在中央电视台组织的"2004中国魅力城市"评选活动中，我市从全国600多个城市中脱颖而出，成为40个入围城市之一，城市风光片在中央电视台多个频道播放，展示了良好的城市形象。中央电视台"东方时空"明星足球队来我市举行了大型义赛义演活动，在省内外引起热烈反响。中国电影表演艺术学会经过考察后，确定今年8月在我市举办中国电影诞辰百年庆典暨第10届金凤凰电影奖颁奖典礼系列活动，国内外近百家新闻媒体进行了宣传报导。先后到济南、郑州、南京等大中城市举行了大规模的旅游和房地产促销活动，赴西安开展了港口·旅游·人居推介活动，并与西安市政府签订了合作协议。这一系列重大活动，大大地提高了日照的知名度，扩大了对外影响，提升了城市形象。我们可以自豪地说，在全市人民的共同努力下，我们的建设和发展越来越为世人所瞩目，一个魅力无限、生机勃勃的新日照正在迅速崛起！

（十）政府自身建设不断加强。一年来，我们以提高行政效率、优化发展环境为目标，大力强化政务监督，取得了良好效果。一是充分发挥行政服务中心集中监督的作用，对行政审批事项实行"一站式"办理、"一条龙"服务，办事效率和服务质量明显提高。二是充分发挥社会监督的作用，大力实施"阳光工程"，建立了政府新闻发布会制度和重大决策征求群众意见制度，开通了"行风在线"直播节目，进一步增强了政府工作的透明度。三是充分发挥专门机关监督的作用，在加强财政、审计等日常监督的同时，借鉴"焦点访谈"的形式，对政府机关和公务人员履行职责、廉洁从政情况进行明察暗访，有力地规范了行政行为。四是充分发挥舆论监督的作用，对人民群众关心的热点、难点公开向社会披露，促进了问题的解决。我市提高政府工作效率的做法，在省政府全体扩大会议上作了介绍。韩国唐津郡郡守来我市考察后，派出7批100多名公务员来我市现场观摩提高行政效率、优化发展环境的经验和做法，这在全省尚属首例。

总之，过去的一年是国家宏观调控力度大、经济发展面临困难多的一年，也是我市大发展、大变化的一年。一年来的实践使我们深切体会到，实现日照的跨越发展，关键是要解放思想，更新观念，坚持以科学发展观统领经济社会全局。

一是必须审时度势、抢抓机遇。去年年初，面对树立落实科学发展观和加强宏观调控的新形势，我们及时召开全市领导干部会议，引导各级认真学习、准确把握科学发展观的内涵，明确提出要把加强宏观调控作为加快发展的大好机遇，坚持做到"八个不动摇"，全力加快日照经济的跨越发展。在半年时我们对全市经济社会发展情况进行了为期三天的现场观摩，强调要以加强执政能力建设为重点，坚定不移地实现跨越发展的目标。全省科学发展情况交流现场会之后，又组织赴潍坊等三市进行了学习考察，开展了"四查四看"活动，从而做到了全年加快发展的目标不变、决心不改、力度不减。去年在国家贷款增量下降的情况下，我市金融机构存贷款余额分别增长21.3%和31.5%，增幅居全省第二位和第一位。地方财政收入提前一个月完成了全年预算任务，全市境内财政总收入实现65.16亿元，增长40.9%。实践充分证明，机遇无处不在、无时不有。只要我们正确把握形势，抢抓发展机遇，就一定能够战胜一切困难险阻，不断夺取新的胜利。

二是必须敢想敢干、勇争一流。2004年我市能够在强手如林、竞争激烈的情况下，入围中国"魅力城市"；能够将中国电影金凤凰奖颁奖和明星足球赛等活动首次争取到地级市来举办；能够与青岛、西安这样的副省级城市签订合作协议，在同一平台上共谋发展；能够开通到西安的旅客列车，圆了全市人民的"通车梦"，都是坚持了敢想敢干、勇争一流的结果。特别是在争取岚山设区的过程中，我们在历届打下的基础上，发扬锲而不舍的精神多次进京做工作，使岚山终于成功设区，成为我省近十年来唯一一个经国务院批准新设的县级行政区，圆了全市人民盼望已久的"设区梦"。事实进一步证明，事在人为。只要我们坚定信心，敢于争先，别人能够办到的事情我们能够办到，别人办不到的事情我们也能办到!

三是必须与时俱进、改革创新。2004年我们把"政府工作创新"活动扩大到全市各级各部门，有效地催化了思想观念和运作方式的更新，使发展中的许多难题迎刃而解。去年全市土地资产经营收益总额16.5亿元，居全省第五位。城市建设80%的资金来自市场化运作，灯塔广场改造、世帆赛基地建设、火车站地下广场等城市建设重点工程，全国沙滩排球赛等大型体育赛事和茶博会、房博会、浮来山福寿文化节等各项会展活动全部实行市场化运作。为了破解企业融资难题，我们以创新的方式成功举办了银企合作促进会，驻鲁25家省级金融机构参加，共有178个项目达成合作意向，签约总额达350亿元，其中贷款合同64个、金额27亿元，到去年底已到位资金24.7亿元。同时，日照港上市已通过证监会发审委审核，昌华集团在新加坡成功上市，结束了我市没有上市公司的历史。各区县、乡镇乃至村居在举办节会、发展园区经济、维护社会稳定等方面也都有改革创新的新举措。实践证明，改革创新出活力、出速度、出效益。坚持改革创新，就能攻坚克难，不断开创经济社会发展的新局面。

四是必须一心为民、真抓实干。一年来，我们把"执政为民"作为一切工作的出发点和落脚点，坚持真心实意地为群众办实事、解难事、做好事，努力让人民群众从改革发展中得到更多的实惠。在农民增收、城市就业、社会保障、困难群体救助等与群众切身利益息息相关的问题上，坚持做到真抓实干，干就干好。年初确定的16件"为民实事"如期完成计划进度，全年新转移农村劳动力7.7万人，新增城镇就业再就业3.7万人；为80个村、4.9万人解决了饮水困难；继续坚持经济适用房改革，为市区572户中低收入家庭解决了住房困难；改造农村中小学校舍3.2万平方米；两县两区全部推行了农村新型合作医疗，受益人口达到98.76万人，区域覆盖率在全省率先达到100%；垃圾处理厂一期工程建成投运；完成农村公路改造1094公里，新增通柏油路村433个；连接市区与岚山的滨海大道开工建设，潮石公路建成通车。这些实事都是经过公开征集确定的，顺民心、合民意，赢得了人民群众的理解、支持和参与。实践证明，只要我们心里装着群众，一切为了群众，紧紧依靠群众，我们的工作就一定能够得到人民群众的支持和拥护，我们的事业就一定能够无往而不胜！

五是必须用心把握、用心工作。针对去年新事多、大事多、难事多的实际，各级坚持把上级指示精神与当地实际相结合，科学判断形势，积极稳妥工作，做到既想干事、会干事，又能干成事、不出事，在实践中锻炼提高了执政能力。坚持综合治理综合治，积极推进"平安日照"建设，保持了治安形势的稳定，成为全省少有的发案总数下降的城市之一。坚持"重心下移、工作前移"，认真解决人民群众来信来访，妥善处理人民内部矛盾，成为全省进京去省上访最少的城市之一。坚持安全责任重于泰山，狠抓安全生产责任制的落实，圆满完成省下达的各项安全指标。特别是在重点建设、旧城改造、海上养殖拆迁等工作中，各级坚持依法办事，精心运作，在加快发展中保持了社会稳定。

各位代表，过去一年我们所取得的成就，是全市各级坚决落实党中央、国务院和省委、省政府各项政策部署、自觉实践科学发展观的结果，是市委正确领导、市人大有效监督支持、市政协积极参政议政的结果，是全市人民同心同德、干事创业的结果，是在历届市领导班子打下的良好基础上取得的。在此，我代表市人民政府，向为日照改革开放和现代化建设做出贡献的全市人民，向所有关心、支持和帮助日照发展的同志们、朋友们，表示崇高的敬意和衷心的感谢!

过去一年取得的成绩令人鼓舞，但前进道路上还有不少困难和问题：经济总量小、结构层次低的问题仍然突出；县域经济发展滞后的问题依然存在；乡镇财政困难的问题还没有得到有效解决；促进农民增收的长效机制尚未建立；社会保障能力比较脆弱，部分低收入群众生活困难；城市化水平不高，特别是"城中村"改造的任务艰巨；一些干部的思想观念和工作水平还不能适应新时期政府工作的要求；一些地方和单位官僚主义作风和腐败现象仍然存在；经济发展的软硬环境都有待进一步改善。对这些困难和问题，我们将高度重视，认真解决。

二、2005年政府工作的任务

2005年是全面实现"十五"计划目标、衔接"十一五"发展的关键一年，也是深入贯彻落实科学发展观、加快日照跨越发展的重要一年。今年政府工作的总体要求是：以邓小平理论和"三个代表"重要思想为指导，深入贯彻党的十六大和十六届三中、四中全会精神，牢牢把握战略机遇期，坚持用科学发展观统领全局，突出跨越发展这一主题，着力抓好结构调整和改革创新"两个关键"，加快推进市场化、城市化、国际化"三化"进程，深入实施港口立市、工业强市、科教兴市、生态建市"四大战略"，努力抓好招商引资、特色经济、民营经济、高新技术和农民增收"五项重点"，大力加强社会主义精神文明和民主法制建设，推动经济社会全面协调可持续发展。具体工作中，要更加注重抓住重要战略机遇期，全力推进跨越发展；更加注重调整经济结构，着力提高经济增长的质量和效益；更加注重发挥市场配置资源的基础性作用，加快体制机制创新；更加注重经济与社会协调发展，促进社会进步和人的全面发展；更加注重坚持以人为本，着力构建社会主义和谐社会。主要预期目标是：生产总值增长15%；地方财政收入增长14%；进出口总值增长27%；人口自然增长率控制在7.2‰以内；城市居民人均可支配收入增长8%；农民人均纯收入增长5%。

为实现上述任务目标，重点抓好八项工作:

（一）高度重视和认真做好"三农"工作。把解决"三农"问题摆到更加突出的位置，按照城乡统筹发展的要求，促进农民增收、农业增效、农村经济社会全面发展。

千方百计增加农民收入。一是继续调整农业和农村经济结构，拓宽农民增收空间。在确保粮食安全的前提下，大力发展茶、果、菜、烟、桑、药、花等特色高效农业，努力把种植业调优。突出发展食草畜禽和规模养殖，努力把畜牧业做大。大力发展水产品工厂化养殖和精深加工，努力把海洋渔业做强。二是加快农业产业化进程，挖掘农民增收潜力。加快培植带动能力强、发展潜力大的农业"龙头"企业，提高农产品加工增值率。积极发展农村合作经济组织和社会化服务体系，不断提高农民进入市场的组织化程度。建立健全农业标准、质量检测、市场监管、疫病防治四大体系，增强农产品的国际竞争力。三是加大农村劳动力转移力度，开辟农民增收的新渠道。坚持走"减少农民、富裕农民"的路子，加强对外出务工的组织、引导、培训和服务，采取就地转移与异地输送相结合的办法，年内新转移农村劳动力5万人以上。

大力发展镇域经济。把培育壮大镇域经济作为解决"三农"问题的重要举措，坚持因地制宜、分类指导，加快发展以工业为主导的镇域经济，尽快改善乡镇财政状况。加强农村小城镇建设和管理，实现产业发展与城镇建设的相互促进。大力实施强镇带动战略，支持优势乡镇率先加快发展，培育一批镇域经济的"龙头"。

全面落实各项农村政策。继续实行粮食直补等政策，年内农业税税率再降低2个百分点。稳定完善农村土地承包，实行严格的耕地保护制度，引导规范土地流转。要加大支农力度，尽快建立稳定的农业投入增长机制。大力推进农业科技进步，加强对农民的培训，进一步提高农业综合生产能力。坚持不懈地抓好农村"水、电、路、医、学"等基础设施建设，不断改善农村生产生活条件，年内基本实现村村通柏油路。

（二）坚定不移地实施"工业强市"战略。把培育"三个一批"作为工业发展的首要任务，不断壮大工业实力，提升整体水平。

努力壮大一批支柱产业。要以培植冶金、粮油加工、液体化工、木制品加工、浆纸、能源六大临港产业和机械、食品、建材、纺织等产业为重点，争上一批投资规模大、带动能力强的项目，加速优势产业规模扩张。要加快钢铁、机械、电力、浆纸、粮油加工等优势产业的发展，围绕优势产业积极发展上下游产品和配套企业，拉长产业链条，培育产业集群，形成集聚效应。继续实施百项重点工业项目，加快传统产业技术改造，全年技改投资确保增长40%以上。

发展一批大企业集团。坚持引进和培植并重，引进一批大项目，培植一批大企业。要加大对日照钢铁、五征集团、森博浆纸等35户重点企业的培育力度，在各方面给予倾斜扶持，优化企业外部环境，促其快速健康发展。年内五征集团销售收入确保过50亿元、争取60亿元，日照钢铁厂确保过80亿元，力争达到100亿元。

培育一批知名品牌。把争创名牌与培植支柱产业、培育大企业集团结合起来，坚持政府引导、企业主导、市场认可、政策激励，运用科技、经济、法律、行政等手段，大力推进质量、安全、环保三个体系认证，加快培育名牌企业和名牌产品，提高我市产品的知名度和市场占有率。年内争创山东名牌产品6个、山东省著名商标3个，中国名牌产品力争实现零的突破。

（三）大力培育特色经济，加快发展服务业。立足我市得天独厚的港路、区位、生态环境优势，大力培育特色鲜明、竞争力强的地方经济品牌，繁荣发展服务业。一是做大做强港口经济。按照建设两个亿吨大港的目标，加大港口基础设施投入力度，着力抓好矿石码头、集装箱码头、石油码头等28项重点工程，打通日照港东西港区，建设口岸通关中心，提高集疏运能力，年内港口货物吞吐量要突破7000万吨。大力发展临港工业，年内临港工业销售收入要翻一番。积极发展港口物流业，加快推进中石化30万吨级原油码头及输油管线项目，规划建设出口加工区、保税区和专业物流园区，完善港口功能。二是发展壮大旅游经济。以滨海旅游资源开发为龙头，以五莲山、浮来山开发为两翼，构筑山海旅游体系。打造旅游精品项目，提升民俗旅游发展水平。加大旅游促销力度，搞好旅游环境整治，推动旅游业再上新台阶。三是大力发展体育经济。办好世界帆船锦标赛、世界帆板锦标赛两项国际体育赛事和全国翻波板锦标赛等11项国家、省级体育赛事，配套完善世帆赛基地设施，努力打造"国际水上运动之都"，使水上运动成为日照对外形象的鲜明标志。四是积极发展房地产经济，打响"日照房产"品牌，吸引更多的外地人来日照购房置业。五是加快发展大学经济。坚持高等教育与科研转化并重，年内新增在校大学生1.5万人，引进科研项目5个。六是改造提升商贸流通业，繁荣城乡市场。特别是按照创特色、上水平和规范管理、便民利民的要求，搞好规划建设，逐步让夜晚亮起来、市场活起来、消费旺起来。同时，积极培育金融保险、信息咨询、中介服务、社区服务等新兴服务业。

（四）全面深化各项改革。大力推进市场化取向的改革，以体制和机制上的比较优势赢得发展上的竞争优势。一是建立健全要素市场体系。大力培育土地、资本、技术、劳动力市场，规范有形建筑市场，鼓励支持中介服务组织发展，充分发挥市场配置资源的基础性作用。完善社会信用体系，推进"诚信日照"建设，努力促进各种要素合理流动、充分竞争。二是大力培育多元化市场主体。按照建立现代企业制度的要求，积极推进所有制结构调整，进一步放宽市场准入，放手发展民营经济。坚持抓环境、抓引进、抓培植、抓提高，一方面大力引进市外民营企业，一方面要做大做强现有的民营企业，加快提升民营经济发展水平，努力实现民营经济的新突破。三是加快推进事业单位改革，年内完成市直经营开发服务类事业单位的改革试点工作。深化行政管理体制改革，进一步健全完善行政服务、行政执法、行政监督"三位一体"的工作体系，推动政府工作提速提效。深化财税体制改革，全面落实政府采购和收支两条线制度，大力推进部门预算，搞好国库集中支付试点，加快构筑公共财政体系。大力培植财源，强化社会综合治税，努力提高财政收入占GDP的比重和税收占财政收入的比重。

（五）加快经济国际化进程。要按照拓宽开放领域、提高招商水平的要求，进一步加大招商引资力度，全年实际外商投资增长50%以上。在招商区域上，坚持国外突破日韩、海外突破港台、省外突破江浙、省内突破青岛，掀起新一轮招商引资热潮。在招商重点上，突出争引工业项目，加快服务业、旅游业、城市建设和农业领域的利用外资。在招商方法上，完善以政府为指导、企业为主体的工作机制，强化专业招商、产业招商、以商招商，提高招商引资成效。要切实发挥园区的示范和载体作用，办好现有各类园区和加工制造业基地，合理规划和用好现有土地，积极探索发展"飞地经济"，努力缓解土地瓶颈制约。牢固树立环境是资源、是竞争力的意识，着力营造高效务实、安商亲民的行政服务环境，诚实守信、遵纪守法的人文社会环境，能够适应外商工作和生活的"仿真"环境，使日照成为最佳创业城市。

（六）进一步加大接轨青岛、融入半岛力度。继续坚持"观念接轨为先导、产业接轨为核心、企业接轨为主体"的思路，进一步拓宽和提升与青岛等半岛城市交流合作的领域与水平。一是加大产业协作力度。按照"传统产业互补、新兴产业共建、协同配套发展"的原则，正确处理竞争与合作的关系，主动承接青岛结构调整中的产业转移，努力把日照建设成为青岛及半岛的大工业配套基地、绿色食品供应基地、休闲度假和旅游观光的后花园。二是做好依托青岛扩大招商引资的文章。加强与驻青岛的国内外企业、金融办事机构的联系，吸引国内外大企业、大集团到日照投资兴业。三是抓好交通联网。积极做好日照至潍坊高速公路前期准备工作，争取尽早开工建设。抓好日照至青岛、日照至岚山的滨海大道建设，使之成为承接青岛及半岛产业转移的黄金大道。

（七）努力提高城市建设管理水平。坚持"三高"要求，加快城市建设，努力把日照建设成为国际水准的滨海城市、富有特色的精品城市。一是进一步完善城市规划。按照"双城一区、轴向拓展"的带形城市发展模式，高起点、高标准完成城市总体规划、土地利用总体规划修编，用一流的规划指导建设。二是抓好重点工程建设。搞好碧海路、北京路等主干道的环境整治和灯塔广场建设、银河公园开放式改造等23项城市建设工程，力争建一项工程、成一件精品。三是不断提高城市管理水平。继续扎实做好创建国家卫生城市和国家环保模范城市工作，深入开展城市环境综合整治活动，下大力气解决"城中村"问题，加强县城、中心镇驻地和农村小城镇建设。四是按照建设全国生态示范市的要求，大力发展循环经济，积极培育生态企业、生态园区、生态产业，搞好生态林业体系建设，保持"蓝天、碧海、金沙滩"的环境优势。（八）努力构建和谐社会。大力加强社会主义精神文明建设，深入开展文明城市创建活动，不断提高人们的思想道德水平和文明程度。继续抓好"平安日照"建设，切实增强人民群众的安全感。进一步搞好国防教育、动员工作，加强国防后备力量建设，做好转业军人和退役士兵转退安置工作，争创"双拥模范城"三连冠。加强农村基层政权建设，抓好村务公开的规范完善，积极推进城市社区建设和管理服务。深入实施"科教兴市"战略，抓好科技成果引进转化，建立健全技术推广和创新体系，积极培育发展高新技术产业。落实教育优先发展的战略地位，完善以县为主的义务教育体制，统筹发展基础教育、高等教育、职业教育、成人教育和民办教育。坚持用好现有人才、引进急需人才、培养未来人才，为跨越发展提供强有力的人才智力保障。积极发展文化、广电事业，加强城市文化建设，活跃农村文化生活。强化疾病预防控制和医疗救治"两个体系"建设，提高城乡医疗保健水平。广泛开展全民健身运动，保障人民身体健康。积极实施可持续发展战略，努力稳定低生育水平，合理开发并有效保护水、土地、海洋、矿产、森林等资源，正确处理经济发展与环境保护的关系，进一步加大环境保护力度，努力实现经济社会与人口、资源、环境的协调发展。

各位代表！重视和关心群众生活，为人民群众排忧解难，是各级政府义不容辞的职责。要坚持在发展中解决就业问题，鼓励发展服务业、劳动密集型产业和中小企业，不断培育新的就业增长点。认真落实就业再就业的各项优惠政策，加强政府就业指导和服务，搞好就业培训，年内新增就业3.2万人，城镇登记失业率控制在3.5%以内。扩大社会保险覆盖面，不断提高保障水平。加强劳动监察，维护劳动者合法权益。加大城乡困难群众救助力度，进一步完善城市居民最低生活保障制度，切实解决农村贫困群众的生活困难问题。继续采取面向群众公开征集的方式确定今年的"为民实事""，并抓好责任分工，认真组织实施，确保落到实处。

三、加强政府自身建设，努力提高执政能力

做好今年的政府工作，必须坚持以提高执政能力为核心，不断加强政府自身建设。要继续按照本届政府在市十五届人大一次会议上向全市人民作出的"四建四有"的承诺，以开展保持共产党员先进性教育活动为契机，进一步提高政府工作水平，更好地履行党和人民赋予的神圣职责。

一是努力提高加快发展的能力。要始终把加快发展作为政府工作的第一要务，真正做到聚精会神搞建设，一心一意谋发展。要科学分析判断形势，始终保持奋发有为、积极向上的精神状态，善于应对挑战，把握机遇，借机加快发展。要不断深化对市场经济规律的认识，提高对事物的认知能力，把握事物的本质规律，努力做到科学发展。要深刻认识和发挥自身的优势，做好上情与下情、机遇与优势的对接，不断开创发展的新局面。要坚持求真务实、真抓实干，工作一经部署，目标一经确定，就要铺下身子狠抓落实，不达目的决不罢休。

二是努力提高改革创新的能力。要深入持久地开展"政府工作创新"活动，大张旗鼓地表彰奖励创新成果，着力营造鼓励创新的浓厚氛围，努力做到以创新促创业、促发展。各级政府要不断创新思想观念和行政方式，坚持一事之前先问市场，凡是能交给市场去办的事情，政府决不包揽；凡是能用经济手段解决的问题，决不用行政手段；凡是能够花钱直接办的事情，决不拿钱"养人"去办。要不断创新发展理念，做到用招商引资的钱发展经济、用市场化运作的钱搞城市建设、用财政的钱改善人民生活。要不断创新体制机制，加快推进事业单位改革，积极创新用人机制，增强内部发展活力。三是努力提高驾驭复杂局势的能力。坚持一手抓正确的导向，做好稳定人心的工作，一手抓综合治理，做好稳定社会的工作。要始终坚持用正确的思想导向凝聚人，努力形成风正气顺心齐、团结和谐稳定、开拓创新发展的局面；用正确的舆论导向引导人，对内唱响干事创业的主旋律，对外树立日照的良好形象；用正确的工作导向激励人，旗帜鲜明地支持创业者，奖励有功者，把干部群众的思想和精力引导到想发展、谋发展、促发展上来。要全面落实社会治安综合治理措施，严厉打击各种违法犯罪活动，认真做好人民来信来访工作，及时化解人民内部矛盾，高度重视安全生产，在加快发展中保持社会稳定。

四是努力提高做群众工作的能力。各级政府领导干部要树立正确的群众观和政绩观，时刻把群众的冷暖疾苦挂在心上，尽心尽力地为群众排忧解难，做人民群众的贴心人。要进一步完善重大决策征求群众意见制度，主动听取各民主党派、工商联、无党派人士、各人民团体和社会各界的意见建议，不断提高政府工作的透明度和公众参与度，赢得群众的理解和支持。要经常深入基层，深入群众，把群众紧紧地凝聚和团结在党和政府的周围，努力形成干群同心、共谋发展的良好局面。

五是努力提高依法行政的能力。政府工作人员特别是领导干部要牢固树立法制观念，严格按照法定权限和程序行使权力，自觉做到依法决策、依法办事。要认真贯彻执行《行政许可法》和《全面推进依法行政实施纲要》，进一步建立健全行政执法责任制，做到有权必有责、用权受监督、违法要追究、侵权要赔偿。要依法接受人大及其常委会的监督，积极支持人民政协履行政治协商、民主监督、参政议政职能，认真办理人大代表议案、建议和政协委员提案。要全面落实廉政建设责任制和廉政准则，严格执行"五个不许"的规定，切实纠正部门和行业不正之风，深入开展反腐败斗争，始终保持为民、务实、清廉的良好形象。

各位代表！

我们在实现跨越发展的征程上已经创造了令人自豪的业绩，但这只是万里长征的第一步，更伟大的事业需要我们去奋斗，更辉煌的业绩需要我们去创造。让我们紧密团结在以胡锦涛同志为总书记的党中央周围，高举邓小平理论伟大旗帜，全面贯彻"三个代表"重要思想，在中共日照市委的正确领导下，解放思想，与时俱进，同心同德，干事创业，为实现跨越发展的宏伟目标而努力奋斗！
